# Variations in Casein Genes Are Associated with Milk Protein and Fat Contents in Sarda Goats (*Capra hircus*), with an Important Role of *CSN1S2* for Milk Yield

**DOI:** 10.3390/ani14010056

**Published:** 2023-12-22

**Authors:** Maria Luisa Dettori, Michele Pazzola, Antonia Noce, Vincenzo Landi, Giuseppe Massimo Vacca

**Affiliations:** 1 Dipartimento Medicina Veterinaria, Università degli Studi di Sassari, 07100 Sassari, Italy; pazzola@uniss.it (M.P.); gmvacca@uniss.it (G.M.V.); 2 Centre de Recerca Agrigenòmica (CRAG), Campus Universitat Autònoma de Barcelona, Universitat Autònoma de Barcelona, 08193 Bellaterra, Spain; noce.antonia.an@gmail.com; 3 Dipartimento Medicina Veterinaria, Università degli Studi di Bari Aldo Moro, 70121 Bari, Italy; vincenzo.landi@uniba.it

**Keywords:** casein gene cluster, sarda goat, capra hircus, dairy goat, milk traits, *CSN1S1*, *CSN2*, *CSN1S2*, *CSN3*

## Abstract

**Simple Summary:**

Extensive goat breeding follows principles of sustainable agriculture, and it can be achieved with local breeds because their genome has been shaped by centuries of interaction with the environment in which goats were raised. The Sarda goat is well adapted to the arid environment of Sardinia (Italy). The aim of the present work was to study the variability of the casein genes, the main proteins of milk. We measured milk traits such as protein, fat, lactose, total solids, and milk yield, and performed association analyses with casein gene variants. The variability of the four casein genes was associated with milk protein, fat, total solids, and milk energy. The main finding was that intronic variants of the *CSN1S2* gene (encoding the αs2-casein protein) were associated with milk yield and protein and fat content. This information might be used in selection schemes, and in future investigations aiming to disclose the direct link between genotype and phenotype.

**Abstract:**

This work aimed to assess the variability of casein genes in a population of 153 bucks and 825 lactating does of the Sarda breed, and to perform association analysis between polymorphic sites and milk yield and composition traits. To genotype the casein genes, we chose an SNP panel including 44 SNPs mapping to the four casein genes *CSN1S1*, *CSN2*, *CSN1S2,* and *CSN3*. Genotyping (made by KASP™ genotyping assay, based on competitive allele-specific PCR) revealed the high variability of the Sarda goat, and haplotype analysis revealed linkage disequilibrium (LD) between *CSN1S1* and *CSN2* genes, in addition to two LD blocks within the *CSN1S2* and two LD blocks within the *CSN3* gene, in bucks and does. Association analysis revealed that variability at all four casein genes was associated with milk protein content, total solids, and milk energy. The three Ca-sensitive casein genes were associated with lipid content, and *CSN1S2* showed a unique pattern, with intron variants associated with milk yield, in addition to milk pH, NaCl, and SCS (Somatic Cell Score). This information might prove useful in selection schemes and in future investigations aiming to better understand the biology of lactation, and the direct link between genotype and phenotype.

## 1. Introduction

Dairy goats provide a reliable source of milk and other dairy products to communities around the world, as they can adapt to different environments, allowing the use of harsh territories that are otherwise unused or abandoned [1,2]. In Europe, the dairy goat sector has evolved into a thriving industry, with dedicated breeds reared under intensive farming systems, and local breeds reared under extensive systems, especially in mountainous areas, drylands, and islands [3]. While cosmopolitan breeds usually have higher milk production capacities when reared under high-input farming systems, the local breeds produce less milk, and they might have good milk quality and aptitude for coagulation [2]. With these premises, the dairy goat sector has the potential to respond to the growing demand for high-quality dairy products, and to meet the consumer preference for sustainable and locally sourced products; goat farming aligns well with the principles of sustainable agriculture [4].

**Figure 1:**
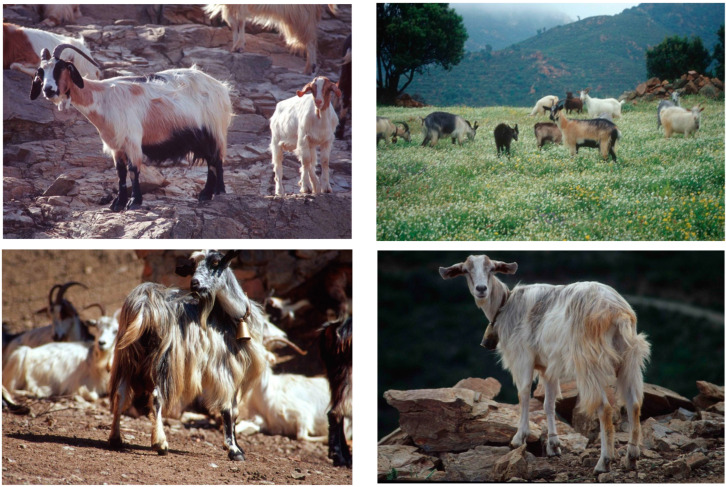
Sarda goats, reared on the island of Sardina.

Sardinia (Italy) is an island located in the center of the Mediterranean Sea, where dairy goat farming is traditionally run under extensive conditions, especially in the central, eastern, and south-western regions [5]. The estimated number of heads of Sarda goats, the local caprine breed reared in Sardinia, is 165,000, of which 25,429 heads are registered in the herdbook of the Sarda breed and make up the official population [6] (Figure 1). The Sarda goat is well adapted to the arid and inaccessible soils of Sardinia, displays high morphological and genetic variability, and its milk is mainly destined for cheese making. The milk yield was reported to be highly variable, about 200 L per lactation, and 0.95 Kg/d for Sarda goats [7].

Caseins make up about 80% of milk proteins, contribute to the nutritional value of milk, and have a functional and structural role, due to their involvement in the formation of casein micelles and milk transformation into cheese. Four different casein proteins are distinguished in goat milk: αs1-casein (encoded by the *CSN1S1* gene), β-casein (encoded by the *CSN2* gene), αs2-casein (encoded by the *CSN1S2* gene), and k-casein (encoded by the *CSN3* gene). The four casein genes form a cluster spanning 250 kbp on chromosome 6 [8]. Casein genes exhibit variability in their genomic DNA sequence, mRNA alternative splicing, transcript-level variation, and post-transcriptional modifications [9,10]. Polymorphisms in casein genes have been shown to affect milk protein and fat contents, milk yield, the organoleptic traits of milk and cheese, coagulation properties, and cheese yield [11]. Leroux et al. (1992) [11] revealed that the *CSN1S1* gene encoding the as1-casein expressed nine different mRNAs, most lacking the three exons 9, 10, and 11. Jansa Perez et al. (1994) [12] demonstrated that the “intermediate” E variant was due to the insertion of a LINE sequence in the 3’UTR region of the *CSN1S1* gene, which destabilized the mRNA, with consequent instability and reduced protein secretion. The molecular basis of casein variants was explained in detail, and numerous studies were conducted to evaluate allelic variants’ occurrence in the different breeds and populations and their association with milk traits. The E variant prevailed in the Saanen and Alpine breeds, followed by F, and the strong allele was *CSN1S1* A [13]. In the Sarda, a local goat breed not subjected to extreme selection, the strong alleles of the *CSN1S1* gene prevailed, with a high frequency of A and B alleles. The occurrence of the B allele associated with a higher expression level than A (both strong alleles) was also highlighted in Spanish goats [14].

In this context, the use of SNPs with a major effect on phenotype highlights the direct effect of the single variation. In contrast, neutral markers serve as reference points distributed in the region of interest, and allow a comparison to identify DNA regions that might be associated with a trait. In the present study, we used a set of neutral markers that ultimately allowed us to highlight the effect of as2-casein on milk yield, which had not been demonstrated so far in the literature. This work aimed to assess the variability of a set of DNA variants in casein genes in a population including bucks and does of Sarda breed, and to perform association analysis between polymorphic sites and milk yield and composition traits. To genotype the casein genes, we chose an SNP panel already tested in Murciano Granadina goats [15].

## 2. Materials and Methods

### 2.1. Animals and Sampling

**Figure 2:**
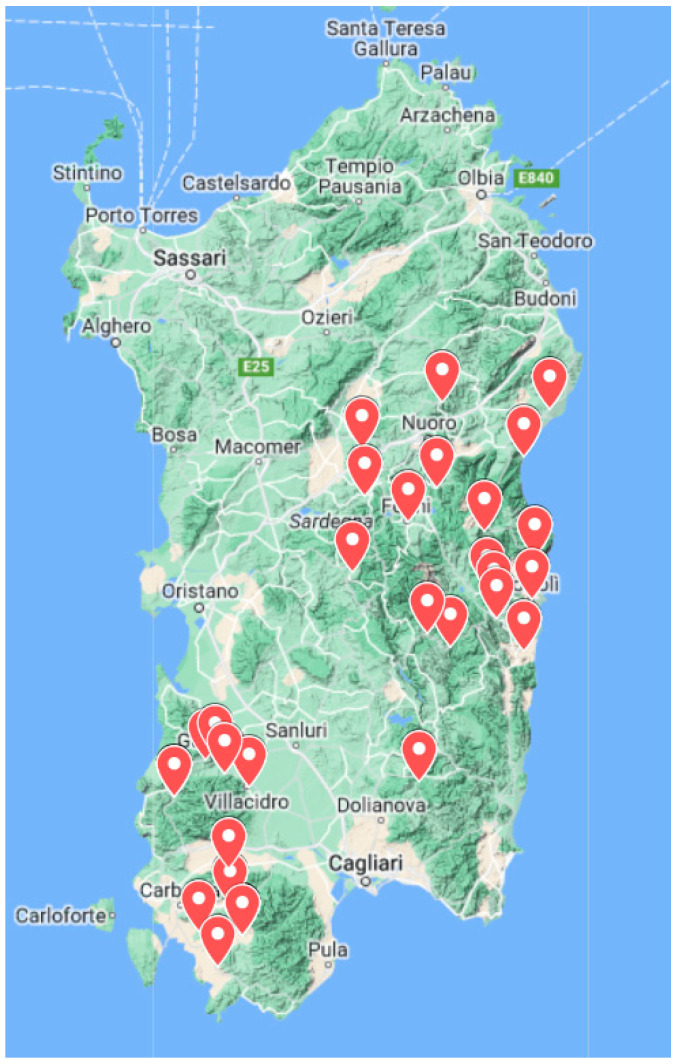
Geographic map showing the distribution of sampling farms in Sardinia. Made with Google MyMaps, https://www.google.com/intl/it/maps/about/mymaps/ (Last accessed on 16 November 2023).

The population analyzed included 825 lactating goats, sampled from 19 farms (about 40 goats per farm), and 153 bucks from 38 farms (1 to 5 per farm). The farms were located in Sardinia’s central, eastern, and south-western areas (Italy) (Figure 2).

Animals were officially registered in the flock books of the Sarda breed and enrolled in the milk recording system of provincial associations of goat breeders. A description of the extensive farming system adopted in Sardinia, and of the most recent historical events that influenced the current composition of the Sardinian goat population, has been given by Usai et al. (2006) [16]. At the same time, a more accurate description of the animals analyzed in this article is reported by Paschino et al. (2020) [5]. Individual milk samples (200 mL/goat) were collected from each female goat in sterile containers during the morning milking and immediately stored at 4 °C; one sampling day was completed on each farm. All the sampled goats were healthy, as certified by a veterinarian. When the milking system was mechanical, milk was taken from the recorder jar of each goat, and the same recorder jar was also used to measure milk yield (MY). When animals were hand-milked, milk was collected from the stainless-steel graduated pail, which was also used to record milk yield. For DNA extraction, a blood sample was taken from each goat in K3EDTA vacuum tubes (BD Vacutainer, Becton Dickinson, Franklin Lakes, NJ, USA).

### 2.2. Phenotyping

Milk samples were analyzed within 24 h after collection. Daily milk yield was calculated as the total record of morning plus evening milkings, measured on the same day of sample collection. Phenotyping included measurements of milk fat, protein, total solids, lactose, pH, and NaCl by using a MilkoScan FT6000 milk analyzer (Foss Electric A/S, Hillerød, Denmark), calibrated according to the following FIL-IDF references: ISO-IDF, 2013 [17], for fat, protein, lactose, pH, and NaCl; ISO-IDF, 2010, for total solids [18]. Milk energy was calculated as the sum of the values of the nutrients, according to the NRC (2001) [19] energy values: fat = 38.89 kJ/g; protein = 23.90 kJ/g; lactose = 16.53 kJ/g. Somatic cell count was evaluated using a Fossomatic 5000 somatic cell counter (Foss Electric) and then transformed into the logarithmic [log2(SCC × 10^−5^) + 3] SCS, according to Shook (1993) [20]. The total bacterial count was evaluated using a BactoScan FC150 analyzer (Foss Electric) and transformed into the logarithmic bacterial count [LBC = log10(total bacterial count/1000)] based on ISO-IDF (2004) [21]. 

### 2.3. Genotyping

Genomic DNA was obtained from leukocytes using the Gentra Puregene Blood Kit (Qiagen, Hilden, Germany), and purity and concentration were measured with an Eppendorf BioPhotometer (Eppendorf, Hamburg, Germany). Forty-four SNPs (Single Nucleotide Polymorphism) spanning the goat casein gene cluster were genotyped in all 978 genomic DNA samples, including 825 does and 153 bucks (Table 1). The 44-SNP panel was designed by Pizarro et al. (2020) [15]. Genotyping was performed with the KASP™ genotyping technology (Kompetitive Allele-Specific PCR assay, LGC Limited, Fordham, UK), and raw allele calls were analyzed with KlusterCaller software (LGC Limited, Fordham, UK).

### 2.4. Bioinformatic and Statistical Analysis

Minor allele frequencies (MAF), observed and expected heterozygosity and the Hardy–Weinberg equilibrium were measured at each polymorphic marker with Haploview v4.2 [22]. Pairwise linkage disequilibrium (LD) measures, including the normalized coefficient of linkage disequilibrium (D′) and the squared correlation (r^2^), as well as haplotype frequencies and the possible occurrence of LD blocks, were estimated with the Haploview v4.2 software package [23]. 

The association analysis between 44 polymorphic marker genotypes and milk traits of 825 does of the Sarda breed was based on the model:

Y = µ + G + F + P + DIM + eijklmn,(1)

where Y is the observed trait; µ is the general mean; G is the fixed effect of the SNP genotype, one at a time (2 to 3 levels: 2 homozygous and the heterozygous); F is the random effect of the farm, which also includes animal management and feeding (1 to 19 levels, representing the different farms included in the experiment); P is the fixed effect of the parity of the goats (1 to 4 levels; first to fourth or more parities); DIM is the fixed effect of the days in milking (4 levels; 1 = ≤100 d; 2 = 101–140 d; 3 = 141–160 d; 4 = ≥161 d); and eijklmn is the random residual effect. 

The same model as in Equation (1) was used to evaluate the association between milk traits and each of the 6 haplotype blocks, one at a time. In the single SNP and LD block analyses, we only considered SNP with an MAF > 0.05 to make sure that genotypic means were correctly estimated. The MIXED procedure of SAS (version 9.4, SAS Institute Inc., Cary, NC, USA) was used to carry out the association analysis. Multiple testing was performed using the Bonferroni method (one milk trait for each SNP or LD block at a time). Model effects were significant at *p* < 0.05.

## 3. Results

### 3.1. Genotyping

All 44 genotyped SNPs were polymorphic in the does population (Table 1), while 39 SNPs were polymorphic in the bucks population (Table S1). The SNPs’ distribution is displayed in Figure S1.

**Table 1:** List of marker mapping to the casein genes, with population parameters, for 825 Sarda goats (*Capra hircus*) reared in Sardinia (Italy).

Gene	#	SNP ID ^1^	Chr_6 Position ^2^	Gene Region	ObsHET ^3^	PredHET ^4^	Hwpval ^5^	MAF ^6^	Alleles
*CSN1S1*	01	rs155505524	85977292	upstream	0.33	0.31	0.17	0.19	A:(G)
	02	CNS1S1_419	85977582	upstream	0.01	0.01	1.00	0.01	T:(G)
	03	rs661930533	85977593	upstream	0.07	0.07	0.16	0.04	C:(G)
	04	rs668059877	85977607	upstream	0.02	0.02	1.00	0.01	A:(G)
	05	rs155505526	85977912	upstream	0.23	0.31	5.0 × 10^−7^	0.19	T:(C)
	06	rs155505527	85977916	upstream	0.33	0.39	3.5 × 10^−5^	0.27	A:(G)
	07	rs664719033	85977985	upstream	0.46	0.48	0.18	0.39	C:(T)
	08	rs155505528	85978133	upstream	0.45	0.50	0.01	0.47	G:(A)
	09	rs155505529	85978197	upstream	0.34	0.39	1.0 × 10^−4^	0.27	A:(G)
	10	rs644189353	85982583	intron_3	0.06	0.07	0.15	0.04	A:(G)
	11	rs663050755	85982740	intron_4	0.34	0.39	8.8 × 10^−5^	0.27	G:(A)
*CSN2*	12	rs658253664	86006394	exon_9	0.45	0.47	0.21	0.39	C:(T)
	13	rs155505539	86008016	exon_7	0.44	0.45	0.68	0.33	T:(C)
	14	rs155505540	86015338	upstream	0.07	0.07	0.67	0.04	A:(G)
	15	rs155505541	86015756	upstream	0.18	0.18	0.85	0.10	G:(A)
	16	rs639868773	86015830	upstream	0.01	0.01	1.00	0.01	G:(A)
	17	rs155505544	86016649	upstream	0.47	0.50	0.20	0.46	C:(T)
*CSN1S2*	18	CNS1S2_451	86075209	upstream	0.20	0.21	0.59	0.12	C:(T)
	19	CNS1S2_437	86075223	upstream	0.47	0.50	0.16	0.48	A:G
	20	CNS1S2_670	86075442	upstream	0.46	0.49	0.05	0.45	G:(T)
	21	CNS1S2_679	86075451	upstream	0.46	0.50	0.05	0.45	T:(C)
	22	CNS1S2_347	86075961	upstream	0.46	0.49	0.03	0.45	G:(A)
	23	rs660278987	86088523	intron_13	0.45	0.46	0.43	0.36	A:(C)
	24	rs268293098	86088596	intron_14	0.35	0.35	0.88	0.22	T:(C)
	25	rs155505547	86088687	intron_14	0.35	0.35	0.73	0.22	C:(G)
	26	rs654867803	86088716	Intron_14	0.45	0.46	0.52	0.36	G:(A)
	27	rs650859238	86088978	Intron_14	0.44	0.46	0.34	0.36	C:(T)
	28	rs666578482	86089078	Intron_14	0.45	0.46	0.46	0.36	A:(G)
	29	rs155505548	86089096	Intron_14	0.17	0.17	0.62	0.10	C:(T)
	30	rs649653184	86089260	intron_14	0.03	0.03	1.00	0.01	A:(G)
	31	rs268293100	86093348	exon_17	0.44	0.46	0.36	0.36	T:(C)
	32	rs638259886	86093378	exon_17	0.17	0.50	4.4 × 10^−87^	0.50	C:(G)
*CSN3*	33	rs155505553	86195919	upstream	0.45	0.45	0.77	0.35	A:(T)
	34	rs669162082	86195968	upstream	0.38	0.39	0.63	0.27	C:(T)
	35	rs155505555	86196117	upstream	0.45	0.45	0.79	0.35	G:(C)
	36	rs155505557	86196315	upstream	0.30	0.34	4.0 × 10^−4^	0.22	T:(G)
	37	rs658757070	86196318	upstream	0.49	0.47	0.28	0.38	C:(T)
	38	rs636747701	86196358	upstream	0.47	0.44	0.08	0.33	T:(C)
	39	rs666872112	86196545	upstream	0.40	0.38	0.44	0.26	C:(T)
	40	rs635706338	86196623	upstream	0.46	0.48	0.24	0.39	A:(T)
	41	rs651457123	86196658	upstream	0.50	0.48	0.37	0.40	AATC:(_)
	42	rs644683886	86196737	upstream	0.39	0.36	0.78	0.24	G:(A)
	43	rs155505560	86196912	upstream	0.39	0.36	0.04	0.24	G:(T)
	44	rs268293114	86209184	Exon_4	0.20	0.23	0.01	0.13	A:(G)

^1^ SNP ID = dbSNP reference records (https://www.ncbi.nlm.nih.gov/projects/SNP/, last accessed on 16 November 2023); ^2^ Chr_6 position = position on chromosome 6, ASM170441v1 Sequence ID—NC_030813.1; ^3^ ObsHET = observed heterozygosity; ^4^ PredHET = predicted heterozygosity; ^5^ HWpval = Hardy–Weinberg test *p*-value. ^6^ MAF = minor allele frequency (minor allele in brackets).

In the does population, at the *CSN1S1* gene, four out of eleven SNPs had minor allele frequency (MAF) lower than 0.05, and four SNPs were out of Hardy–Weinberg Equilibrium (HWE). At *CSN2*, all SNPs were in HW equilibrium, in the *CSN1S2* gene only 1 SNP out of 15 was highly significant for HWE, while in the CSN3 gene, 3 SNPs were out of HWE (Table 1). In the bucks population, five SNPs overall were monomorphic and only one SNP of the *CSN1S2* gene did not follow Hardy–Weinberg Equilibrium (rs638259886) (Supplementary Table S1). The DNA polymorphic markers used in the present investigation fell mostly in the upstream DNA region of the four casein genes, although some were in intronic regions and 3’UTR (*CSN1S2* gene). All markers were SNPs (Single Nucleotide Polymorphism) except one indel (insertion-deletion), rs651457123 at the CSN3 gene, and two missense-variants, rs155505539 at *CSN2* exon 7, and rs268293114 at CSN3 exon 4. Both missense variants were predicted to be tolerated by the SIFT prediction algorithm (http://sift.jcvi.org/, last accessed on 10 November 2023).

### 3.2. Descriptive Statistics and Association Analysis

The descriptive statistics of daily milk yield and quality traits of 825 Sarda does are shown in Supplementary Table S2. The most variable phenotypes were LBC and milk yield (MY), with coefficients of variation of 48% and 45%, respectively. 

The results from the ANOVA (F-values) of daily milk yield and quality traits versus 44 SNP genotypes mapped to the casein genes are reported in Table S3. The SNPs with significant effects on phenotype variance are detailed in Table 2, and the effects of haplotype blocks are reported in Table 3.

At *CSN1S1*, two out of eleven SNPs were significantly associated with milk protein content (*p* < 0.001), rs664719033 and rs155505528, and these were also associated with fat content (*p* < 0.05 and *p* < 0.01, respectively). The same SNPs showed association with milk NaCl content (*p* < 0.05), and their impact on milk traits extended to total solids and milk energy traits (*p* < 0.001) (Table 2). At the *CSN2* gene, the SNPs rs658253664 and rs155505544 were associated with milk protein content at *p* < 0.001 and with the fat content at *p* < 0.01; the same SNPs were associated with total solids and milk energy (Table 2). In addition, SNPs rs1555505539 and rs155505541 were associated with milk protein content at *p* < 0.01. All the above-mentioned SNPs were associated with NaCl content. SNP rs155505539 at the *CSN2* gene was associated with SCS at *p* < 0.01. At the *CSN1S2* gene, 10 out of 15 genotyped SNPs were highly associated with milk protein content (*p* < 0.001), and 4 SNPs were associated with milk fat content (*p* < 0.01); this association was also reflected in milk total solids and milk energy (Table 2). In total, 6 SNPs at *CSN1S2* were associated with the milk yield trait (*p* < 0.05) and 10 SNPs were associated with milk pH (*p* < 0.05); moreover, 6 SNPs were associated with the SCS trait (*p* < 0.01) and 6 SNPs were associated with milk NaCl content (Table 2). At the CSN3 gene, 3 out of 12 genotyped SNPs were associated with milk protein content at *p* < 0.001, and 2 SNPs were associated with protein content at *p* < 0.01. Furthermore, only two SNPs were associated with fat content (*p* < 0.05) and showed contextual association with milk energy (*p* < 0.01). Finally, CSN3 SNP rs658757070 was associated with milk NaCl content.

### 3.3. LD Analysis

**Figure 3:**
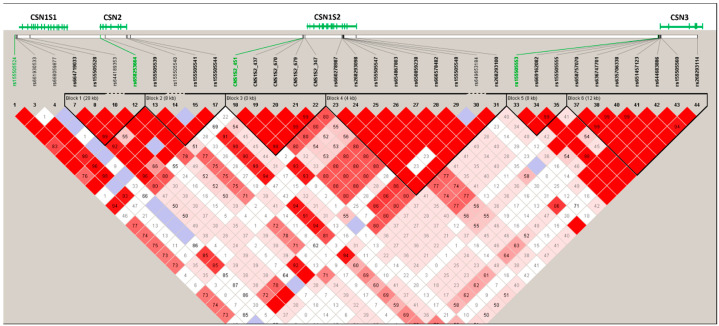
LD structure of the casein gene cluster DNA region in a population of 825 Sarda does. The structural organization of the four casein genes respects gene positions on goat chromosome 6, as represented in Genome Data Viewer (https://www.ncbi.nlm.nih.gov/genome/gdv/?org=capra-hircus, last accessed on 18 November 2023). LD plot of pairwise normalized coefficient of linkage disequilibrium (D′): coloured in red, D′ = 1.0 and logarithm of the odds (LOD) ≥ 2.0; coloured in light blue, D′ = 1.0 and LOD < 1.0.

**Figure 4:**
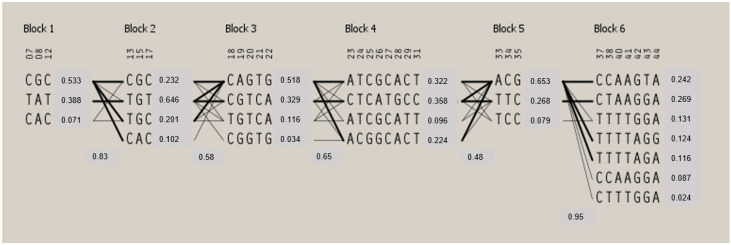
Haplotype blocks defined by the 44 SNPs genotyped in the four casein genes in 825 Sarda does. Haplotype frequencies are reported near each haplotype within a block. For each crossing area, a value of the multiallelic normalized coefficient of linkage disequilibrium (D′) is given for the haplotypes displayed, which represents the level of recombination between the two blocks. SNP numbering is reported in Table 1.

Linkage disequilibrium (LD) analysis was performed on doe (Figure 3 and Figure 4) and buck (Figures S2 and S3) populations.

After the exclusion of SNPs out of HWE and those with MAF values lower than 0.05, the casein cluster’s haplotype structure included 35 SNPs for does and 38 SNPs for bucks, spanning about 231 kbp, with an average distance between SNPs of 5.3 kb, ranging from 3 bp to 102 kbp. Block 1 (28 kb) in the doe population included two tag SNPs of the *CSN1S1* gene located in the promoter region and one SNP of the *CSN2* gene located in exon 9 (Figure 1). The same structure was visible in the buck population, in which block 2 included three SNPs from the *CSN1S1* gene and two SNPs from the *CSN2* gene. The *CSN1S2* and *CSN3* genes displayed two haplotype blocks each in both populations. In both populations, block 6 consisted of six SNPs, and had seven different haplotype combinations, while block 1 and block 5 comprised three SNPs, and had three possible haplotype combinations (Figure 3 and Figure S1).

## 4. Discussion

The genotyping of 44 SNPs at casein genes, in a sample population of 825 does and 153 bucks, revealed the high variability of the Sarda breed goat, as all analyzed SNPs were polymorphic, and of these 15% were rare (MAF < 0.05). Bucks also displayed 9% monomorphic SNPs, probably due to a different selection pressure on males and females, related to milk production, and to the effects of positive and purifying selection on nucleotide variation and diversity [24].

In Sarda goats, genotyping of the functional allele variants at the four casein genes revealed the occurrence of all investigated alleles in does and bucks, including rare defective variants such as *CSN1S1* E (with a frequency of 0.03) and null alleles, only at Ca-sensitive casein genes, with frequencies lower than 0.01 Vacca 2014 [25]. The high variability of Sarda goats is probably due to the strategies adopted by the breeders on the island [26]. In addition to these strategies to maintain variability, farmers have also made, in the past, unplanned crosses with exotic breeds to increase milk production, but this proved to be a counterproductive practice in the long run [16]. The variability of casein cluster genes and structure has been reported in livestock species [27,28,29,30] and in other species, such as donkeys [31] camelids [32,33], elephants [34,35] and llamas [36], contributing to the reconstructing phylogenesis of the DNA region and bringing new insights into the casein function and formation of casein micelles (reviewed in [37]).

### 4.1. Association Analysis—The Outstanding Role of CSN1S2

In the present study, we revealed an association of casein gene variants with milk protein and fat content. This association extends to milk total solids and milk energy. The association of the casein genes’ variability with milk protein and lipid content has been reported in different goat breeds [38,39], and it has also been highlighted in other dairy species, such as sheep [40,41] and cattle [42,43]. Martin et al. (2017) [44], in a GWAS study, found that the casein cluster region was associated with protein content and fat content in French goats. Massender et al. (2023) [45] found that casein genes were of key economic importance in Canadian Saanen and Alpine goats, and revealed the presence of several candidate positional and functional SNPs in the region.

The SNP panel used in the present investigation allowed us to highlight, in this context, the outstanding role played by the *CSN1S2* gene as regards its association with milk yield (MY), in addition to the association with milk protein content, shared among the four casein genes, and the association with fat content, shared among the Ca-sensitive caseins genes. SNPs associated with MY were located in the *CSN1S2* gene body, at introns 13 and 14 and exon 17. That association might be due to LD with an unknown causal mutation, or to unknown biological pathways, which still need to be elucidated. The *CSN1S2* gene is phylogenetically the most recent in the casein cluster [13], and variation in this gene might have given a selective advantage in Sarda dairy goats. MY is a polygenic quantitative trait, of high economic and productive importance, and more studies are needed to better understand the biological mechanisms that regulate this type of productive trait. The association of *CSN1S2* gene variants with milk fat and total solid concentration has been revealed in Chinese goats (Yue 2013 [46]), with daily milk yield and milk coagulation parameters in Sarda goats [47] and milk protein and casein contents in sheep [41]; no association between *CSN2* and *CSN1S2* genes and milk protein or dry matter contents were found in Murciano Granadina goats [48]. The *CSN1S2* gene was associated with Somatic Cell Score (SCS) in the present study, another critical parameter, considered an indirect indicator of milk hygienic quality, important in the physiology of lactation in goats [49,50].

### 4.2. CSN1S1 and CSN2 Genes Are Associated with Milk Protein and Fat Content

The *CSN1S1* and *CSN2* genes are convergently transcribed and are just 10.9 kbp apart on goat chromosome 6. Linkage Disequilibrium (LD) analysis showed the occurrence of a haplotype block including SNPs rs664719033, mapping to the *CSN1S1* upstream region, and rs658253664, mapping to the *CSN2* exon 9 (block 1) in the does population. Similarly, LD block 2 included SNPs from the *CSN1S1* and *CSN2* genes in bucks. The two genes have a total of four SNPs associated with milk protein and lipid content, and consequently also with total solids and milk energy.

In French dairy goats, the *CSN1S1* gene has been reported to explain about 40% of protein content variation, and its genotype can be used as a positional and functional candidate to better predict genomic breeding values [51]. Different alleles of the αs1-casein gene have been reported to affect milk protein content, protein yield, fat content, and milk yield [13,52] in dairy goats. In addition, polymorphism of the caprine *CSN1S1* gene has been reported to affect the technological properties of milk, such as cheese yield and cheese curd formation [53].

Variants at the *CSN1S1* gene have been associated with four different levels of as1-casein in milk; in Sarda goats, the *CSN1S1* B allele was reported to be the most frequent and was associated with an as1-casein content in milk of 4.94 g/L per allele [54], higher than the values reported for the *CSN1S1* A variant [14]. The *CSN1S1* F “weak” allele had a frequency of 0.25, and the remaining defective alleles were rare [25]. The defective alleles of as1-casein can influence in different ways the correct formation of casein micelles, causing variations in the morphology of the mammary gland epithelial cells [39]. Some authors do not detect any effect of casein variants *CSN1S1* and *CSN3* on the physicochemical characteristics of Saanen goat milk [55]. Some studies have revealed an effect of *CSN2* on milk quality traits and milk renneting properties, except for milk yield, in Sarda goats [53].

### 4.3. CSN3 Variation Is Associated with Protein Content

The *CSN3* gene showed a highly significant association with proteins, as did all the casein genes. Weak associations were found with lipid content, total solids, and milk energy. This is in accordance with the structural role played by k-casein in stabilizing casein micelles, and with a fundamental role in the coagulation processes that occur in the newborn’s stomach or during transformation processes aimed at cheesemaking [56]. The variability of this gene in goat breeds has been reported [57]. Association analysis in Sarda goats revealed a highly significant effect of variation at the *CSN3* gene on milk coagulation parameters [53]. Susilorini et al. (2022) [58] indicated an association between *CSN3* gene polymorphism and milk yield and composition in indigenous Indonesian goats.

Rahmatalla et al. (2021) [59] analyzed the casein cluster in search of new rare variants in both wild and farmed Indian goats, and revealed that most novel SNP variants occurred in the endangered Nubian ibex. The genetic variability of local breeds has been shaped over time in their environment, and represents a precious reservoir of biodiversity that should be protected. Although local breeds, such as the Sarda goat, are less productive than transboundary breeds, they are adapted to the local climate. They can be reared under extensive farming systems in a natural environment [60].

## 5. Conclusions

Variability of the Sarda does at casein genes was associated with milk protein content. In addition, *CSN1S1* and *CSN2*, which were in linkage disequilibrium, were also associated with the lipid content, and then with milk total solids and milk energy. The *CSN1S2* gene differed from the other genes because, in addition to its association with milk protein and fat content, it also showed an association with milk yield and other milk traits (pH value, SCS, NaCl). The *CSN3* gene variants were associated with milk protein content. This information might be used in selection schemes and might guide future investigations to better understand lactation’s biology, potentially disclosing the direct link between genotype and phenotype.

## Figures and Tables

**Table 2:** Least square means of Sarda goat milk traits according to the different genes, SNPs and genotypes (n = 825).

Gene	SNP	Genotype	n	MY	Fat %	Protein %	Lactose %	Total Solids	pH	SCS	LBC	NaCl	Milk Energy
*CSN1S1*	rs155505526	CC	33	0.97	5.16 ^ab^	4.23	4.78	15.15 ^ab^	6.76	5.81	1.84	213.5	3.81 ^ab^
		TC	96	1.04	5.43 ^a^	4.22	4.76	15.40 ^a^	6.73	5.78	1.84	212.6	3.91 ^a^
		TT	294	1.03	5.08 ^b^	4.11	4.77	14.94 ^b^	6.73	5.70	1.85	214.9	3.75 ^b^
	rs664719033	CC	301	1.02	5.62 ^a^	4.09 ^A^	4.64	15.31 ^A^	6.71	6.41	1.70	229.6 ^b^	3.93 ^A^
		TC	363	1.04	5.41 ^b^	3.97 ^B^	4.64	15.02 ^B^	6.72	6.60	1.66	237.3 ^ab^	3.82 ^B^
		TT	134	1.06	5.28 ^b^	3.87 ^C^	4.63	14.74 ^B^	6.72	6.35	1.58	242.7 ^a^	3.74 ^B^
	rs155505528	AA	196	1.07	5.22 ^B^	3.90 ^C^	4.64	14.72 ^B^	6.72	6.43	1.60	240.8 ^a^	3.73 ^B^
		GA	362	1.04	5.48 ^A^	3.99 ^B^	4.64	15.10 ^A^	6.72	6.56	1.65	235.6 ^ab^	3.85 ^A^
		GG	249	1.02	5.63 ^A^	4.09 ^A^	4.64	15.32 ^A^	6.71	6.45	1.72	230.3 ^b^	3.94 ^A^
*CSN2*	rs658253664	CC	312	1.02	5.61 ^A^	4.09 ^A^	4.64	15.29 ^A^	6.71	6.41	1.70	230.1 ^b^	3.93 ^B^
		TC	364	1.04	5.41 ^AB^	3.97 ^B^	4.64	15.02 ^B^	6.72	6.61	1.66	237.2 ^ab^	3.82 ^B^
		TT	129	1.07	5.25 ^B^	3.86 ^C^	4.63	14.72 ^C^	6.73	6.36	1.57	242.3 ^a^	3.73 ^B^
	rs155505539	CC	93	1.01	5.59	4.10 ^A^	4.66	15.27	6.71	6.02 ^B^	1.60	226.5 ^b^	3.91
		TC	353	1.03	5.49	4.03 ^AB^	4.64	15.13	6.71	6.57 ^B^	1.71	233.5 ^ab^	3.86
		TT	362	1.06	5.40	3.94 ^B^	4.63	14.98	6.72	6.52 ^B^	1.63	239.0 ^a^	3.82
	rs155505541	AA	9	0.89	5.62 ^ab^	4.28 ^AB^	4.67	15.53 ^A^	6.69	6.03	1.82	216.8 ^b^	3.98 ^A^
		GA	144	1.02	5.70 ^a^	4.07 ^A^	4.65	15.38 ^AB^	6.71	6.41	1.69	228.4 ^ab^	3.95 ^A^
		GG	651	1.05	5.42 ^b^	3.98 ^B^	4.64	15.00 ^B^	6.72	6.52	1.65	237.1 ^a^	3.82 ^B^
	rs155505544	CC	240	1.02	5.64 ^A^	4.08 ^A^	4.65	15.31 ^A^	6.71	6.31	1.68	229.0 ^B^	3.93 ^A^
		TC	377	1.03	5.43 ^B^	4.00 ^A^	4.63	15.06 ^AB^	6.71	6.64	1.69	236.6 ^A^	3.84 ^B^
		TT	180	1.07	5.30 ^B^	3.90 ^B^	4.63	14.80 ^B^	6.73	6.51	1.58	241.8 ^A^	3.76 ^B^
*CSN1 S2*	CNS1S2_437	AA	224	1.02	5.60	4.09 ^A^	4.64	15.31 ^A^	6.71	6.32	1.67	230.9	3.93 ^A^
		GA	380	1.04	5.41	4.00 ^AB^	4.63	14.99 ^B^	6.72	6.58	1.67	237.7	3.82 ^B^
		GG	198	1.07	5.42	3.90 ^B^	4.66	14.99 ^B^	6.73	6.53	1.63	236.0	3.81 ^B^
	CNS1S2_670	GG	259	1.01	5.60	4.09 ^A^	4.63	15.29 ^A^	6.71 ^a^	6.40	1.67	232.2	3.93 ^a^
		TG	371	1.04	5.41	3.99 ^B^	4.63	14.98 ^B^	6.72 ^b^	6.57	1.67	236.8	3.82 ^B^
		TT	176	1.06	5.39	3.89 ^B^	4.66	14.97 ^B^	6.73 ^a^	6.48	1.63	235.9	3.79 ^B^
	CNS1S2_679	CC	176	1.07	5.41	3.89 ^B^	4.66	14.98 ^B^	6.73 ^a^	6.46	1.63	236.1	3.80 ^B^
		TC	371	1.04	5.40	3.99 ^B^	4.63	14.98 ^B^	6.72 ^b^	6.59	1.67	237.2	3.82 ^B^
		TT	259	1.01	5.59	4.09 ^A^	4.63	15.29 ^A^	6.71 ^b^	6.40	1.67	232.2	3.92 ^A^
	CNS1S2_347	AA	174	1.07	5.43	3.90 ^b^	4.66	15.01	6.73	6.48	1.63	235.9	3.81 ^b^
		GA	365	1.05	5.40	3.99 ^ab^	4.64	14.98 ^a^	6.72	6.59	1.67	236.9	3.82 ^ab^
		GG	263	1.01	5.58	4.08 ^a^	4.63	15.27 ^b^	6.71	6.37	1.67	232.2	3.92 ^a^
	rs660278987	AA	338	1.00 ^b^	5.60 ^A^	4.08 ^A^	4.64	15.30 ^A^	6.71 ^b^	6.29 ^B^	1.68	230.9 ^B^	3.93 ^A^
		CA	358	1.07 ^a^	5.36 ^B^	3.97 ^B^	4.62	14.92 ^B^	6.71 ^b^	6.72 ^A^	1.66	240.0 ^A^	3.80 ^B^
		CC	108	1.07 ^a^	5.37 ^AB^	3.87 ^B^	4.68	14.93 ^AB^	6.74 ^a^	6.43 ^B^	1.61	233.5 ^B^	3.78 ^B^
	rs268293098	CC	38	1.01	5.69	4.07	4.62	15.53	6.70 ^b^	6.65 ^ab^	1.66	230.3	3.98
		TC	279	1.02	5.50	4.04	4.61	15.12	6.71 ^b^	6.71 ^a^	1.68	237.9	3.87
		TT	485	1.05	5.43	3.97	4.66	15.02	6.73 ^a^	6.38 ^b^	1.65	234.1	3.83
	rs155505547	CC	481	1.05	5.43	3.97 ^b^	4.65	15.02	6.72 ^a^	6.39 ^b^	1.65	234.2	3.83
		GC	281	1.01	5.51	4.04 ^a^	4.61	15.13	6.71 ^b^	6.70 ^a^	1.69	237.6	3.88
		GG	37	1.02	5.68	4.06 ^a^	4.65	15.52	6.71 ^b^	6.35 ^b^	1.59	227.1	3.98
	rs654867803	AA	104	1.07 ^a^	5.35 ^B^	3.87 ^C^	4.69 ^a^	14.91 ^A^	6.74 ^a^	6.37 ^B^	1.61	232.5 ^B^	3.77 ^B^
		GA	351	1.07 ^a^	5.34 ^B^	3.96 ^B^	4.62 ^b^	14.89 ^A^	6.71 ^b^	6.70 ^A^	1.66	239.8 ^A^	3.79 ^B^
		GG	330	1.00 ^b^	5.60 ^A^	4.08 ^A^	4.64 ^ab^	15.30 ^B^	6.71 ^b^	6.26 ^B^	1.69	230.9 ^B^	3.93 ^A^
	rs650859238	CC	337	1.00 ^b^	5.61 ^A^	4.08 ^A^	4.64	15.30 ^A^	6.71 ^b^	6.29 ^B^	1.68	230.9 ^a^	3.93 ^A^
		TC	356	1.07 ^a^	5.36 ^B^	3.97 ^B^	4.62	14.92 ^B^	6.72 ^ab^	6.71 ^A^	1.67	239.7 ^b^	3.80 ^B^
		TT	110	1.06 ^a^	5.37 ^B^	3.87 ^C^	4.68	14.91 ^B^	6.74 ^a^	6.49 ^AB^	1.60	234.3 ^ab^	3.78 ^B^
	rs666578482	AA	336	1.00 ^b^	5.59 ^a^	4.08 ^A^	4.64	15.29 ^A^	6.71 ^b^	6.28 ^B^	1.68	231.0 ^b^	3.92 ^A^
		GA	357	1.07 ^a^	5.36 ^b^	3.96 ^B^	4.62	14.92 ^B^	6.72 ^b^	6.71 ^A^	1.67	239.9 ^a^	3.80 ^B^
		GG	108	1.07 ^b^	5.36 ^b^	3.87 ^B^	4.68	14.91 ^B^	6.74 ^a^	6.47 ^AB^	1.60	234.1 ^b^	3.78 ^B^
	rs268293100	CC	109	1.07 ^a^	5.37 ^b^	3.87 ^B^	4.68	14.92	6.74 ^a^	6.44 ^AB^	1.61	234.0 ^a^	3.78 ^B^
		TC	355	1.07 ^a^	5.36 ^b^	3.97 ^AB^	4.62	14.92	6.71 ^b^	6.72 ^A^	1.66	239.8 ^a^	3.80 ^B^
		TT	335	1.00 ^b^	5.60 ^a^	4.07 ^A^	4.64	15.29	6.71 ^b^	6.27 ^B^	1.67	230.8 ^b^	3.92 ^A^
	rs638259886	CC	336	1.09 ^a^	5.41 ^B^	3.95 ^B^	4.64	15.00 ^AB^	6.70 ^a^	6.65 ^AB^	1.65	236.6 ^AB^	3.81 ^AB^
		CG	132	1.07 ^a^	5.31 ^B^	3.98 ^B^	4.57	14.87 ^B^	6.68 ^b^	6.83 ^A^	1.69	244.6 ^A^	3.77 ^B^
		GG	334	1.02 ^b^	5.66 ^A^	4.09 ^A^	4.62	15.38 ^A^	6.68 ^b^	6.21 ^B^	1.69	230.9 ^B^	3.94 ^A^
*CSN3*	rs669162082	CC	432	1.03	5.55 ^a^	4.05 ^A^	4.64	15.23 ^A^	6.72	6.46	1.67	233.4	3.90 ^A^
		TC	306	1.06	5.36 ^b^	3.96 ^B^	4.64	14.93 ^B^	6.72	6.49	1.64	236.6	3.80 ^B^
		TT	61	1.08	5.44 ^ab^	3.92 ^B^	4.65	14.93 ^B^	6.71	6.69	1.66	234.8	3.81 ^B^
	rs658757070	CC	307	1.06	5.38	3.92 ^B^	4.64	14.90 ^b^	6.72	6.65	1.65	238.5 ^a^	3.80 ^b^
		TC	391	1.02	5.50	4.03 ^A^	4.63	15.14 ^ab^	6.71	6.45	1.68	235.5 ^ab^	3.87 ^a^
		TT	105	1.04	5.53	4.12 ^B^	4.67	15.28 ^a^	6.73	6.32	1.65	227.1 ^b^	3.90 ^a^
	rs635706338	AA	276	1.06	5.39	3.93 ^B^	4.64	14.93 ^b^	6.72	6.62	1.65	238.4	3.81
		TA	331	1.03	5.49	4.01 ^AB^	4.64	15.09 ^ab^	6.72	6.44	1.67	235.3	3.86
		TT	120	1.02	5.56	4.12 ^A^	4.66	15.30 ^a^	6.72	6.43	1.65	229.6	3.92
	rs651457123	AA	279	1.07	5.40	3.92 ^B^	4.64	14.93 ^b^	6.72	6.63	1.66	238.9	3.81
		AT	398	1.03	5.49	4.01 ^AB^	4.63	15.11 ^ab^	6.71	6.45	1.67	235.0	3.86
		TT	123	1.02	5.56	4.12 ^A^	4.66	15.30 ^a^	6.73	6.41	1.65	228.9	3.92
	rs644683886	AA	43	1011	5.57 ^a^	4.23 ^A^	4.59	15.39 ^a^	6.70	6.36	1.67	231.9	3.94 ^A^
		GA	293	1037	5.58 ^a^	4.06 ^AB^	4.63	15.27 ^ab^	6.69	6.42	1.74	233.3	3.91 ^A^
		GG	462	1073	5.44 ^b^	3.97 ^B^	4.63	15.02 ^b^	6.69	6.58	1.64	237.5	3.83 ^B^
	rs155505560	GG	453	1.02	5.48	4.02	4.65	15.13	6.72	6.36 ^b^	1.65	233.0	3.86
		TG	314	1.07	5.42	3.98	4.62	14.97	6.71	6.69 ^a^	1.67	238.9	3.82
		TT	35	1.01	5.61	3.92	4.62	15.21	6.73	6.33 ^b^	1.74	236.3	3.92

For each SNP, least squares means with different superscripts are different. Capital letters = *p* < 0.01; lowercase letters = *p* < 0.05.

**Table 3:** Least square means of Sarda goat milk traits according to the different haplotype blocks.

Gene	SNP	Genotype	n	MY	Fat %	Protein %	Lactose %	Total Solids	pH	SCS	LBC	NaCl	Milk Energy
Haplotype	Block 1	CGC	245	0.99	5.53	4.10 ^A^	4.65	15.22 ^a^	6.71	6.52	1.73	232.7	3.89 ^a^
Blocks		TAT	125	1.05	5.28	3.87 ^B^	4.63	14.74 ^b^	6.73	6.23	1.58	240.6	3.74 ^b^
	Block 2	TGT	177	1.05	5.28	3.86 ^b^	4.63 ^b^	14.75	6.73	6.37	1.52	243.8 ^a^	3.74
		CGC	45	1.11	5.49	4.06 ^a^	4.69 ^a^	15.15	6.75	6.27	1.45	228.2 ^b^	3.88
	Block 3	CAGTG	222	1.02	5.59	4.09 ^A^	4.64	15.30	6.71 ^b^	6.34	1.65	229.9	3.93
		CGTCA	100	1.01	5.46	3.89 ^B^	4.65	15.05	6.74 ^a^	6.44	1.62	236.1	3.81
	Block 4	ATCGCACT	101	1.04	5.50	4.04 ^a^	4.66	15.16	6.71 ^b^	5.95	1.61	230.2	3.88
		CTCATGCC	101	1.03	5.38	3.90 ^b^	4.68	14.93	6.74 ^a^	6.46	1.64	232.9	3.78

For each SNP, least squares means with different superscripts are different. Capital letters = *p* < 0.01; lowercase letters = *p* < 0.05.

## Data Availability

The data presented in this study are available on request from the corresponding author.

## Supplementary Materials

The following supporting information can be downloaded at: https://www.mdpi.com/article/10.3390/ani14010056/s1, Figure S1: Distribution of SNPs on Casein genes; Figure S2: LD structure of the casein gene cluster in a population of 153 Sarda bucks; Figure S3: Description of haplotype blocks in 153 Sarda bucks; Table S1: List of SNPs of 153 Sarda bucks, mapping to the casein genes, with population parameters; Table S2: Descriptive statistics of milk yield and composition of 825 milk samples of Sarda goats; Table S3: Analysis of variance (*F*-value and significance) of each SNP for milk yield and composition of 825 Sarda does milk samples.
