# Supplementary material for: Variations in Casein Genes Are Associated with Milk Protein and Fat Contents in Sarda Goats (Capra hircus), with an Important Role of CSN1S2 for Milk Yield
Source: Animals (Basel). 2023 Dec 22;14(1):56. doi: 10.3390/ani14010056 (PMC10778555; doi:10.3390/ani14010056)
Supplement: Supplementary file 1 [file animals-14-00056-s001.zip › animals-2671458-supplementary.pdf]

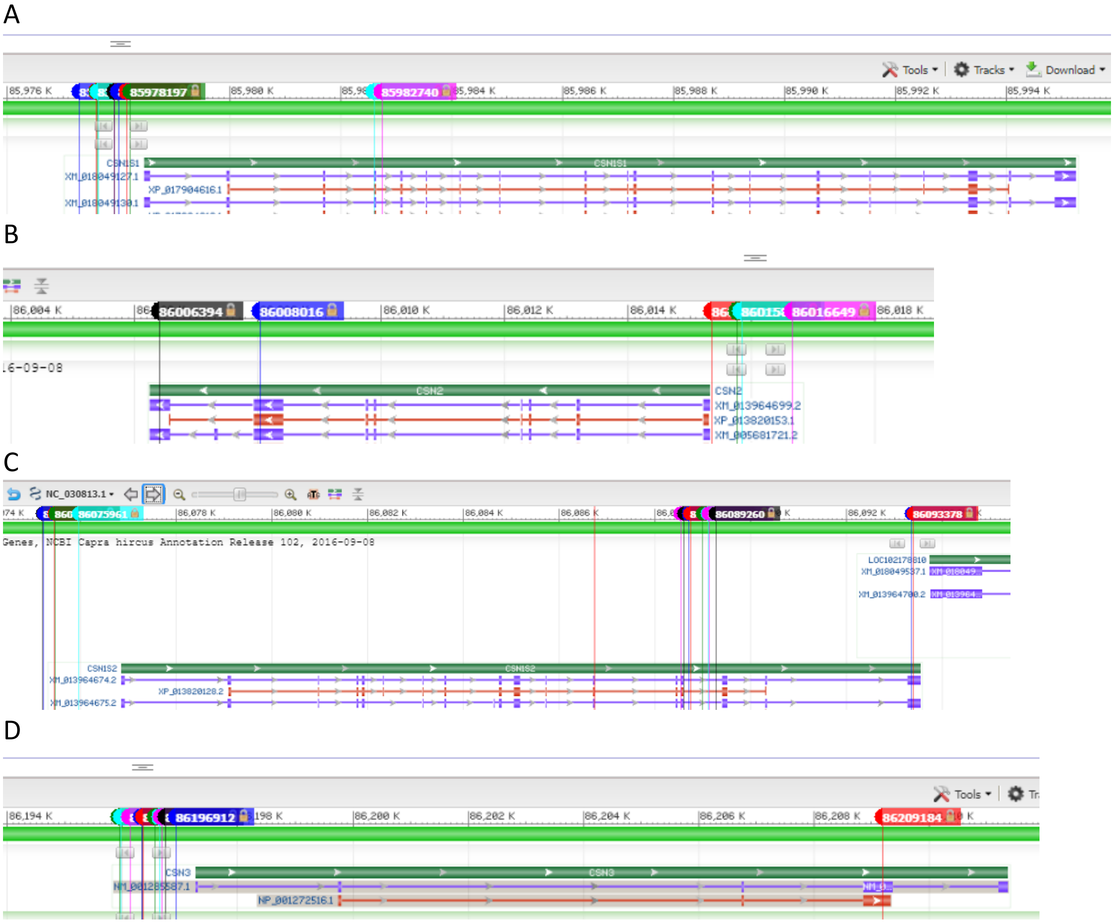

Figure S1. Distribution of SNPs on Casein genes, shown as colored tags, as retrieved from the Genome Data Viewer (https://www.ncbi.nlm.nih.gov/genome/gdv/?org=capra-hircus). Horizontal green bars represent the gene sequence. Horizontal purple bars represent mRNA. Vertical purple bars represent exon regions. A, CSN1S1. B, CSN2. C, CSN1S2. D, CSN3

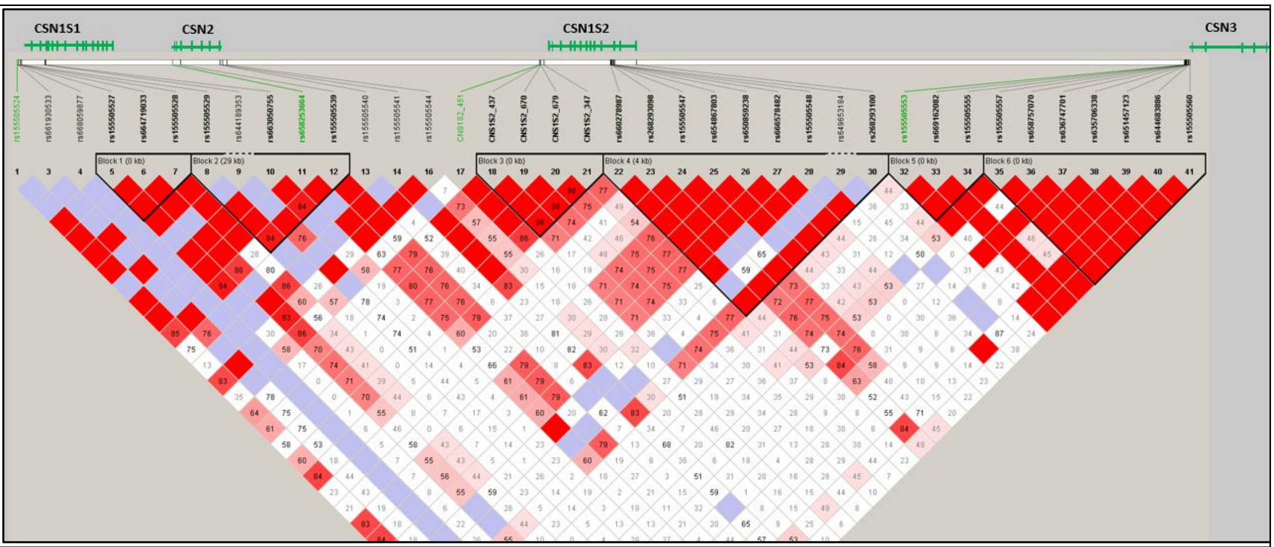

Figure S2. LD structure of the casein gene cluster in a population of 153 Sarda bucks. The structural organization of the four casein genes respects positions on goat chromosome 6, as represented in Genome Data Viewer (https://www.ncbi.nlm.nih.gov/genome/gdv/?org=capra-hircus, last accessed on 18 November 2023). LD plot of pairwise normalized coefficient of linkage disequilibrium (D′): coloured in red, D′ = 1.0 and logarithm of the odds (LOD) ≥2.0; coloured in light blue, D′ = 1.0 and LOD < 1.0 and LOD

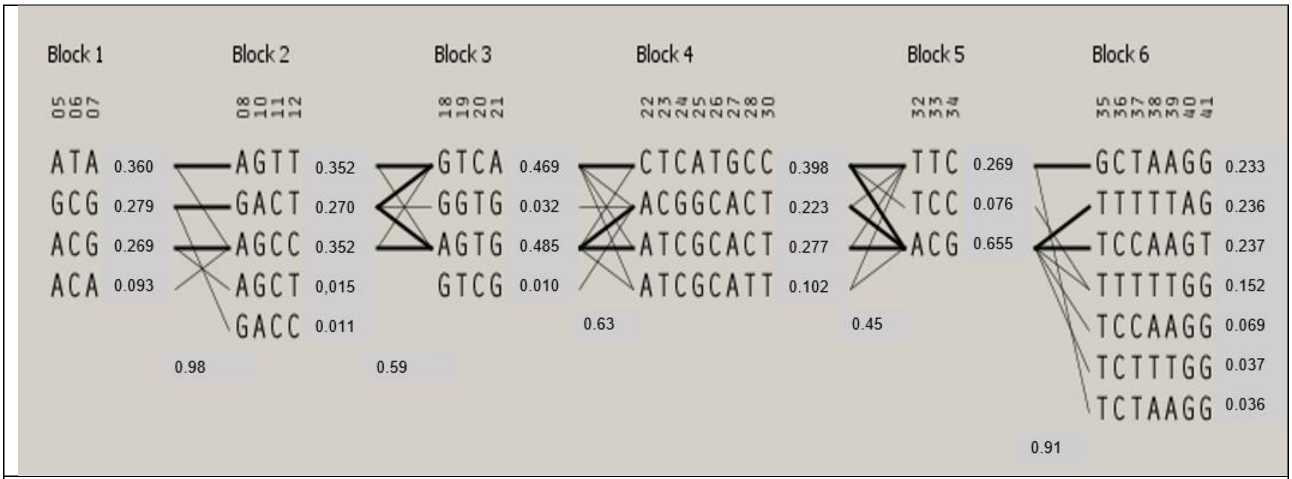

Figure S3. Description of haplotype blocks in 153 Sarda bucks. Haplotype frequencies are reported near each haplotype within a block. For each crossing area, a value of multiallelic normalized coefficient of linkage disequilibrium (D′) is given for the haplotypes displayed, which represents the level of recombination between the 2 blocks. SNP numbers are reported in Table 1

Table S1. List of SNPs of 153 Sarda bucks, mapping to the casein genes, with population parameters.

Gene	#	SNP ID	ObsHET	PredHET	Hwpval	MAF	Alleles
CSN1S1	01	rs155505524	0.32	0.32	1.00	0.20	A:(G)
	03	rs661930533	0.10	0.10	1.00	0.05	C:(G)
	04	rs668059877	0.01	0.01	1.00	0.01	A:(G)
	06	rs155505527	0.37	0.40	0.53	0.28	A:(G)
	07	rs664719033	0.49	0.45	0.39	0.35	C:(T)
	08	rs155505528	0.49	0.50	1.00	0.45	G:(A)
	09	rs155505529	0.39	0.41	0.82	0.29	A:(G)
	10	rs644189353	0.10	0.09	1.00	0.05	A:(G)
	11	rs663050755	0.39	0.41	0.67	0.28	G:(A)
CSN2	12	rs658253664	0.50	0.45	0.33	0.35	C:(T)
	13	rs155505539	0.49	0.46	0.57	0.36	T:(C)
	14	rs155505540	0.12	0.11	1.00	0.06	A:(G)
	15	rs155505541	0.13	0.15	0.43	0.08	G:(A)
	17	rs155505544	0.52	0.49	0.62	0.44	C:(T)
CSN1S2	18	CNS1S2_451	0.18	0.23	0.04	0.13	C:(T)
	19	CNS1S2_437	0.52	0.50	0.75	0.48	G:(A)
	20	CNS1S2_670	0.53	0.50	0.64	0.48	G:(T)
	21	CNS1S2_679	0.52	0.50	0.70	0.49	T:(C)
	22	CNS1S2_347	0.49	0.50	1.00	0.47	G:(A)
	23	rs660278987	0.45	0.48	0.61	0.40	A:(C)
	24	rs268293098	0.34	0.34	1.00	0.22	T:(C)
	25	rs155505547	0.33	0.34	0.78	0.22	C:(G)
	26	rs654867803	0.45	0.48	0.58	0.40	G:(A)
	27	rs650859238	0.46	0.48	0.62	0.40	C:(T)
	28	rs666578482	0.45	0.48	0.61	0.39	A:(G)
	29	rs155505548	0.19	0.18	1.00	0.10	C:(T)
	30	rs649653184	0.02	0.02	1.00	0.01	A:(G)
	31	rs268293100	0.45	0.48	0.58	0.40	T:(C)
	32	rs638259886	0.16	0.49	7.9E-17	0.44	C:(G)
CSN3	33	rs155505553	0.48	0.45	0.52	0.34	A:(T)
	34	rs669162082	0.39	0.39	0.95	0.27	C:(T)
	35	rs155505555	0.47	0.45	0.86	0.34	G:(C)
	36	rs155505557	0.35	0.35	0.92	0.23	T:(G)
	37	rs658757070	0.48	0.48	1.00	0.39	C:(T)
	38	rs636747701	0.40	0.42	0.62	0.30	T:(C)
	40	rs635706338	0.48	0.49	0.91	0.42	A:(T)
	41	rs651457123	0.51	0.49	0.66	0.42	AATC:(_)
	42	rs644683886	0.37	0.36	0.94	0.23	G:(A)
	43	rs155505560	0.33	0.36	0.48	0.24	G:(T)

[1] SNP ID = dbSNP reference records (https://www.ncbi.nlm.nih.gov/projects/SNP/)

[2] ObsHET = observed heterozygosity

[3] PredHET = predicted heterozygosity

[4] HWpval = Hardy-Weinberg test P-value.

[5] MAF = minor allele frequency (minor allele in brackets)

Table S2. Descriptive statistics of milk yield and composition of 825 milk samples of Sarda goat.

Trait	Mean	SD	Min	Max	CV %
MY	1.05	0.47	0.12	3.00	45
Fat	5.50	1.38	1.37	9.94	25
Protein	3.99	0.48	2.77	5.54	12
Lactose	4.64	0.26	3.81	5.33	6
Total solids	15.10	1.69	11.33	20.16	11
pH	6.72	0.10	6.41	7.02	2
NaCl	235.0	49.4	98.0	389.7	21
SCS	6.52	1.81	2.11	11.06	28
LBC	1.66	0.80	0.30	4.23	48
Milk energy	3.86	0.60	2.30	5.66	16

SD, standard deviation; CV %, coefficient of variation

Table S3. Analysis of variance (F-value and significance) of each SNP for milk yield and composition of 825 Sarda does milk samples.

	SNP ID	MY	Fat	Protein	Lactose	Total solids	pH	SCS	LBC	NaCl	Milk energy
CSN1S1	rs155505524	0.7	1.0	1.8	2.7	0.5	1.7	0.2	0.0	0.4	1.2
	CSN1S1_419	2.7	0.0	0.0	0.2	0.0	0.1	0.0	0.2	0.4	0.0
	rs661930533	0.2	1.4	0.1	0.7	1.1	1.7	0.2	0.2	0.2	1.3
	rs668059877	0.0	0.1	1.0	1.2	0.8	1.1	3.1	1.8	1.5	0.4
	rs155505526	0.8	3.8*	2.8	0.1	4.5*	1.9	0.1	0.0	0.1	4.4*
	rs155505527	2.7	0.7	0.3	0.1	0.5	0.4	1.6	1.2	0.2	1.1
	rs664719033	0.7	4.5*	13.8***	0.1	7.4***	1.0	1.6	1.9	4.6*	7.4***
	rs155505528	1.0	6.9**	10.7***	0.0	9.8***	1.0	0.5	2.2	3.1*	9.8***
	rs155505529	2.6	0.6	0.5	0.0	0.4	0.2	1.5	1.2	0.0	0.9
	rs644189353	0.6	1.3	0.2	0.9	1.2	1.7	0.3	0.2	0.5	1.3
	rs663050755	2.7	0.6	0.4	0.2	0.5	0.4	1.6	1.0	0.3	1.0
CSN2	rs658253664	0.9	4.8**	13.5***	0.0	7.4***	1.7	1.7	2.3	4.1*	7.5***
	rs155505539	1.0	0.9	5.2**	0.3	2.2	0.9	4.3**	1.5	2.7*	1.2
	rs155505540	0.6	0.4	0.3	0.4	0.7	2.1	0.0	0.1	0.1	0.5
	rs155505541	1.4	4.6*	6.1**	0.2	5.8**	1.6	0.6	0.7	4.0*	5.3**
	rs639868773	2.6	0.0	0.0	0.2	0.0	0.1	0.0	0.2	0.4	0.0
	rs155505544	1.4	5.0**	8.8***	0.5	6.7**	1.1	2.8	2.4	4.9**	6.3**
CSN1S2	CNS1S2_451	1.4	0.3	1.4	2.8	0.5	1.8	0.7	1.2	1.9	0.5
	CNS1S2_437	1.2	2.7	12.0***	1.1	5.0**	2.1	1.9	0.4	2.2	4.9**
	CNS1S2_670	1.3	3.0	12.8***	1.3	5.1**	3.4*	0.9	0.4	1.1	5.5**
	CNS1S2_679	1.5	2.8	12.7***	1.0	5.0**	3.4*	1.1	0.3	1.3	5.2**
	CNS1S2_347	1.6	2.5	11.2***	0.7	4.4*	2.8	1.4	0.4	1.1	4.6*
	rs660278987	3.8*	5.2**	13.8***	2.7	7.8***	4.2*	6.1**	0.8	4.8**	8.3***
	rs268293098	1.1	1.3	2.8	2.9	2.6	4.2*	3.3*	0.3	1.2	2.3
	rs155505547	1.1	1.2	3.4*	2.9	2.7	3.9*	3.3*	0.8	1.6	2.2
	rs654867803	4.4*	5.9**	13.5***	3.3*	8.7***	4.2*	6.4**	0.8	4.8**	9.3***
	rs650859238	3.5*	5.1**	13.6***	2.2	8.1***	3.7*	5.5**	0.9	4.3*	8.4***
	rs666578482	3.7*	4.6*	13.4***	2.5	7.5***	3.7*	5.9**	0.8	4.5*	7.8***
	rs155505548	0.2	0.6	0.7	0.3	1.1	0.8	0.4	1.7	0.5	0.8

	rs649653184	0.0	0.0	0.8	0.0	0.3	0.0	0.4	0.0	1.9	0.3
	rs268293100	3.2*	3.6*	8.8***	1.7	1.8	3.3*	4.6**	1.5	3.3*	5.6***
	rs638259886	4.1*	6.8**	11.1***	2.4	9.9***	3.0*	5.8**	0.5	5.0**	10.1***
CSN3	rs155505553	1.2	2.4	1.5	1.3	2.2	0.0	0.3	0.6	0.6	3.3*
	rs669162082	1.1	3.2*	6.1**	0.0	5.2**	0.2	0.4	0.3	0.6	4.8**
	rs155505555	1.0	2.6	1.4	1.2	2.3	0.0	0.4	0.6	0.6	3.5*
	rs155505557	0.2	0.9	2.5	0.6	2.0	1.2	1.2	1.6	1.8	1.7
	rs658757070	1.0	1.5	12.9***	1.2	4.3*	1.6	2.0	0.3	3.3*	3.3*
	rs636747701	0.4	0.0	2.3	0.4	0.2	0.2	1.9	0.3	1.0	0.1
	rs666872112	2.2	2.6	2.5	0.4	2.7	0.8	0.1	1.0	0.1	2.9
	rs635706338	1.0	1.2	10.6***	0.3	3.5*	0.3	1.0	0.2	2.1	2.6
	rs651457123	1.5	1.2	11.2***	0.7	3.7*	0.9	1.2	0.0	2.8	2.8
	rs644683886	1.9	3.1*	6.7**	0.1	4.1*	1.8	1.1	2.4	1.2	4.6**
	rs155505560	1.9	0.8	1.8	2.2	1.6	2.7	3.9*	0.5	2.1	1.5
	rs268293114	1.4	2.0	1.7	0.2	1.8	1.2	0.4	1.5	0.2	2.0
Haplotype Blocks	Block 1	1.1	2.2	13.6***	0.4	5.4*	0.7	1.5	2.8	1.5	4.5*
	Block 2	0.3	1.0	3.3*	3.9*	2.1	1.4	0.3	0.5	4.3*	1.4
	Block 3	0.0	0.8	15.1***	0.0	1.9	6.4*	0.2	0.1	1.5	3.6
	Block 4	0.0	0.5	5.1*	0.3	1.6	4.2*	3.8	0.2	0.2	1.7
	Block 5	0.2	0.7	3.3	1.8	2.0	0.1	0.0	0.5	0.6	1.6

* = P < 0.05; ** = P < 0.01; *** = P < 0.001
